# ﻿Three new taxa of the lichen genus *Lobothallia* (Megasporaceae, Ascomycota) from China

**DOI:** 10.3897/mycokeys.108.126994

**Published:** 2024-09-16

**Authors:** Yanyun Zhang, Lun Wang, Xinmeng Yu, Su Cheng, Junlan Liu, Xinyu Wang

**Affiliations:** 1 College of Life Sciences, Anhui Normal University, 241000, Wuhu, China; 2 Yunnan Key Laboratory for Fungal Diversity and Green Development, Kunming Institute of Botany, CAS, 650201, Kunming, China; 3 Key Laboratory of Phytochemistry and Natural Medicines, Kunming Institute of Botany, CAS, 650201, Kunming, China

**Keywords:** New species, new variety, Qinghai-Tibetan Plateau, saxicolous lichen, taxonomy

## Abstract

Two new species; *Lobothalliacrenulata* Lun Wang & Y. Y. Zhang, *L.lobulata* Lun Wang & Y. Y. Zhang and one new variety; L.subdiffractavar.rimosa Lun Wang & Y. Y. Zhang, are reported from China and described, based on morphological, chemical and molecular characters. Phylogenetic analyses showed that these new taxa form monophyletic groups. *Lobothalliacrenulata* and *L.lobulata*, together with *L.hydrocharis*, *L.radiosa* and *L.recedens*, form a well-supported clade, whereas L.subdiffractavar.rimosa is nested within the samples of *L.subdiffracta*. *Lobothalliacrenulata* is characterised by its placodioid thallus, thickly pruinose upper surface with a rimose appearance, aspicilioid to lecanorine apothecia with a crenate thalline margin and concave, black and pruinose discs. *Lobothallialobulata* is characterised by its placodioid thallus, pruinose upper surface with lobules, aspicilioid when immature, lecanorine to zeorine apothecia at maturity and concave to plane, dark brown, shiny and epruinose discs. Lobothalliasubdiffractavar.rimosa is characterised by its areolate thallus, rimose and pruinose upper surface, lecanorine apothecia and slightly concave to plane, black and pruinose discs. Secondary metabolites were not detected in the two new species nor the new variety. A key is provided for the species of *Lobothallia* in China.

## ﻿Introduction

*Lobothallia* (Clauzade & Cl. Roux) Hafellner was originally established as a subgenus within *Aspicilia* A. Massal. (Clauzade and Roux 1984), then later treated as a distinct genus by Hafellner (1991). The genus *Lobothallia* is characterised by its immersed to adnate, or constrictively sessile apothecia, an epihymenium N– reaction to slightly greenish, an algal layer below the hypothecium, a non-amyloid ascus tholus (*Aspicilia*-type), with shorter ascospores (< 18 µm) and conidia (< 8 µm) compared to other genera of the family Megasporaceae (Nordin et al. 2010; Kou et al. 2013; Paukov et al. 2019).

Initially, *Lobothallia* was established to accommodate four marginally lobate species; including *L.alphoplaca* (Wahlenb.) Hafellner, *L.melanaspis* (Ach.) Hafellner, *L.praeradiosa* (Nyl.) Hafellner and *L.radiosa* (Hoffm.) Hafellner (Hafellner 1991). Subsequently, multiple species were transferred into *Lobothallia*, based on their phylogeny and taxonomy. These additions included lobate species: *Aspiciliahydrocharis* Poelt & Nimis, *Lecanorahedinii* H. Magn., *L.platycarpa* J. Steiner (Nimis 2016; Paukov et al. 2019; Zulfiqar et al. 2022) and non-lobate or vaguely lobate species, such as *Aspiciliafarinosa* (Flörke) Flagey, *A.recedens* (Taylor) Arnold and *Lecanorasubdiffracta* H. Magn. (Nordin et al. 2010; Paukov et al. 2019). To date, the genus includes 28 species worldwide (https://www.indexfungorum.org/).

Ten species of *Lobothallia* have been reported from China: *L.alphoplaca*, *L.cheresina* (Müll. Arg.) A. Nordin, Cl. Roux & Sohrabi, *L.crassimarginata* Kou & Q. Ren, *L.hedinii* (H. Magn.) Paukov, A. Nordin & Sohrabi, *L.praeradiosa*, *L.pruinosa* Kou & Q. Ren, *L.radiosa*, *L.semisterilis* (H. Magn.) Y. Y. Zhang, *L.subdiffracta* (H. Magn.) Paukov and *L.zogtii* Paukov & Davydov (Magnusson 1940, 1944; Reyim et al. 2012; Kou et al. 2013; Paukov et al. 2019; Wei 2020; Zhang et al. 2020). Secondary metabolites have been reported for all of these species except, *L.subdiffracta*. From 2014 to 2022, we conducted several field surveys of lichen across the Qinghai-Tibetan Plateau Region, during which, ca. 100 specimens were collected of the genus *Lobothallia*. Several of these specimens differed from the known species in their morphology, molecular phylogeny and absence of secondary metabolites. Here, we describe two new species and one new variety within the genus *Lobothallia*.

## ﻿Materials and methods

### ﻿Morphological and chemical examination

In this study, 108 specimens were examined and deposited in the following Herbaria: Anhui Normal University (AHUB), Lichen Herbarium, Kunming Institute of Botany, Chinese Academy of Sciences (KUN-L) and Shandong Normal University (SDNU). The external morphological characters of air-dried material were studied under a stereomicroscope (OLYMPUS SZ61TR). Anatomical features were studied using a light microscope (OLYMPUS BX43) on transverse sections of apothecia and thalli, prepared manually with a razor blade and mounted in water or lactophenol cotton blue (LCB). Spore measurements were presented as: (minimum–) (x̄ - SD) – x̄ – (x̄ + SD) (–maximum), where x̄ is the arithmetic mean and SD is the standard deviation (values were rounded to the nearest 0.5 µm), followed by the number of measurements (n) (Li et al. 2023). Lugol’s solvent (I) was used to examine the apical structure of asci. Crystals in apothecia and thallus were observed in polarised light (POL) and their solubility was assessed in 10% potassium hydroxide (KOH) (K). Spot tests were conducted using K and a saturated aqueous solution of sodium hypochlorite (NaClO) (C). Secondary metabolites were analysed using thin layer chromatography (TLC) with the solvent C (Orange et al. 2001).

### ﻿DNA extraction, PCR and sequencing

Genomic DNA was extracted from dry or fresh specimens using the DNAsecure Plant Kit (Tiangen, China), according to the manufacturer’s instructions. The fungal internal transcribed spacer (ITS) region and mitochondrial small subunit (mtSSU) of rDNA were amplified using the primers, ITS1F (5′ CTTGGTCATTTAGAGGAAGTAA 3′) (Gardes and Bruns 1993), ITS4a (5′ CGCCGTTACTGGGGCAATCCCTG 3′) (Larena et al. 1999), mrSSU1 (5′ AGCAGTGAGGAATATTGGTC 3′) and mrSSU3r (5′ ATGTGGCACGTCTATAGCCC 3′) (Zoller et al. 1999). Amplifications were performed in a 25 μl volume containing 12.5 μl 2 × Trio Taq Master Mix (Monad Anhui), 1 μl of each primer, 9.5 μl ddH_2_O and 1 μl DNA. PCR-cycle conditions were: initial denaturation at 94 °C for 5 min, followed by 30 cycles of 94 °C for 15 s, 53 °C for 15 s and 72 °C for 1 min and a final extension at 72 °C for 10 min. The PCR products were visualised on 1% agarose gels. The PCR products were sequenced by GENERAL Biosystems (Chuzhou, China) using the amplification primers.

### ﻿Phylogenetic analyses

The raw sequences were initially checked with the BLAST tool on the NCBI online service (https://blast.ncbi.nlm.nih.gov/Blast.cgi) to confirm the lichen affinity. Geneious v.8.0. was used to assemble and edit the raw sequences and generate a single matrix for nrITS and mtSSU. Each matrix was aligned using the MAFFT v.7 online server (https://mafft.cbrc.jp/alignment/server/). Before concatenating the single-gene matrices of nrITS and mtSSU, we tested for potential incongruity using IQ-TREE with 1000 ultrafast bootstrap replicates. No well supported conflict was detected. SequenceMatrix 1.7.8 (Vaidya et al. 2011) was used to concatenate the nrITS and mtSSU genes and produce a 2-locus dataset. PartitionFinder v.2.0 (Lanfear et al. 2017) was used to estimate the best schemes and nucleotide substitution models for Maximum Likelihood (ML) and Bayesian Inference (BI) analyses. The best-fit models for ITS1, 5.8S, ITS2 and mtSSU were GTR+G.

Phylogenetic relationships were inferred using Bayesian Inference and Maximum Likelihood. The Bayesian method was performed with MrBayes 3.2.7 (Ronquist et al. 2012), using four Markov chains running for 12 million generations. Trees were sampled every 100 generations and the first 25% were discarded as burn-in. Subset rates were modelled as fixed and equal. We used the default distributions for priors. The average standard deviation of split frequencies fell below 0.01 by the end of the analysis. Tracer v.1.7 (Rambaut et al. 2018) was used to assess chain convergence by checking the effective sampling size (ESS > 200). ML analyses were performed with RaxmlGUI (Silvestro and Michalak 2012) using the general time reversible model of nucleotide substitution with the gamma model of rate heterogeneity (GTRGAMMA). All trees were visualised using Mega v.7.0 (Kumar et al. 2016) and edited using PowerPoint. Bayesian posterior probabilities ≥ 0.95 and ML bootstrap values ≥ 70% were presented on the ML tree.

## ﻿Results and discussion

The nrITS-mtSSU data matrix encompassed a total of 91 sequences (61 nrITS, 30 mtSSU, including 49 downloaded from GenBank and 42 newly generated) from 61 samples of 22 taxa (Table 1). The length of the final aligned dataset was 1342 nucleotides. Three species, *Aspiciliacinerea* (L.) Körb., *Circinariaesculenta* (Pall.) Sohrabi and *C.fruticulosa* (Eversm.) Sohrabi were chosen as the outgroup, following previous phylogenetic studies (Nordin et al. 2010; Paukov et al. 2019).

**Table 1.** Sequences used in the phylogenetic analyses in this study, with specimen information and GenBank accession numbers. Newly-obtained sequences are in bold font. “na” indicates that there is no sequence available.

Species	Country	Voucher specimens	GeneBank accession number		Reference
			nrITS	mtSSU	
*Aspiciliacinerea*	Sweden: Dalarna	Hermansson 13275 (UPS)	EU057899	HM060695	Nordin et al. (2010)
*Circinariafruticulosa*	Russia: Chelyabinsk	Paukov 3074 (UFU)	MK347508	MK348227	Paukov et al. (2019)
*C.esculenta*	Kazakhstan: Kyzylorda	UFU L-1743	MK347507	MK348226	Paukov et al. (2019)
*Lobothalliaalphoplaca*	Norway	O-L-200411	MK812484	na	Marthinsen et al. (2019)
*L.alphoplaca*	Ukraine: Donetzk	SK A20	KT456207	KT456211	Kondratyuk et al. (2015)
*L.alphoplaca*	China: Inner Mongolia	Tong 20117616 (SDNU)	JX499233	na	Kou et al. (2013)
*L.brachyloba*	Russia: Republic of Altai	Frolov 357 (UFU) Holotype	MK347506	MK348228	Paukov et al. (2019)
*L.crenulata*	China: Xizang	ZYY22-331 (AHUB)	**PP663141**	**PP663164**	This paper
*L.crenulata*	China: Xizang	ZYY22-301 (KUN-L) Holotype	**PP663142**	**PP663165**	This paper
*L.cheresina*	Greece	Sipman & Raus 63224 (B)	MN172423	na	Unpublished
*L.crassimarginata*	China: Inner Mongolia	Wang 20122565 (SDNU) Holotype	JX476026	na	Kou et al. (2013)
*L.crassimarginata*	China: Inner Mongolia	Tong 20122583 (SDNU)	KC007439	na	Kou et al. (2013)
*L.densipruinosa*	Pakistan	LAH 36790 Holotype	MZ871507	na	Ashraf et al. (2022)
*L.densipruinosa*	Pakistan	LAH 36951	MZ871515	na	Ashraf et al. (2022)
*L.elobulata*	Pakistan	LAH 37153 Holotype	ON384441	na	Zulfiqar et al. (2022)
*L.elobulata*	Pakistan	LAH 37154	ON428667	na	Zulfiqar et al. (2022)
*L.epiadelpha*	Russia: Orenburg	Paukov 1881 (UFU) Holotype	MK347505	MK348232	Paukov et al. (2019)
“*L.helanensis*”	China: Inner Mongolia	Tong 20122517 (SDNU) Holotype	JX476030	na	Kou et al. (2013)
“*L.helanensis*”	China: Inner Mongolia	Tong 20122791 (SDNU)	JX476031	na	Kou et al. (2013)
*L.hydrocharis*	Italy: Sardinia	JN72085b (BOLO)	OQ073922	na	Nascimbene et al. (2023)
*L.hydrocharis*	Italy: Sardinia	SMNS-STU-F-0002807 (STU)	OQ073923	na	Nascimbene et al. (2023)
*L.iqbalii*	Pakistan	LAH 37149 Holotype	ON384444	na	Zulfiqar et al. (2022)
*L.iqbalii*	Pakistan	LAH 37150	ON384445	na	Zulfiqar et al. (2022)
*L.lobulata*	China: Sichuan	ZYY22-819 (KUN-L) Holotype	**PP663143**	**PP663166**	This paper
*L.lobulata*	China: Sichuan	ZYY22-822 (AHUB)	**PP663144**	**PP663167**	This paper
*L.lobulata*	China: Sichuan	Wang et al. 22-73395 (KUN-L)	**PP663145**	**PP663168**	This paper
*L.lobulata*	China: Sichuan	Wang et al. 22-73396 (KUN-L)	**PP663146**	**PP663169**	This paper
*L.lobulata*	China: Sichuan	ZYY22-829 (AHUB)	**PP663147**	**PP663170**	This paper
*L.lobulata*	China: Sichuan	ZYY22-824 (AHUB)	**PP663148**	**PP663171**	This paper
*L.melanaspis*	Sweden: Jämtland	Nordin 6622 (UPS)	HQ259272	HM060688	Nordin et al. (2011)
*L.melanaspis*	Norway	Owe-Larsson 8943a (UPS)	JF825524	na	Valadbeigi et al. (2011)
*L.pakistanica*	Pakistan	LAH 37137 Holotype	ON392718	na	Zulfiqar et al. (2022)
*L.pakistanica*	Pakistan	LAH 37139	ON392720	na	Zulfiqar et al. (2022)
*L.praeradiosa*	Russia: Orenburg	UFU L-1264	MK347501	MK348229	Paukov et al. (2019)
*L.praeradiosa*	China: Xinjiang	Huang 20126355 (SDNU)	JX499230	na	Kou et al. (2013)
*L.praeradiosa*	China: Xinjiang	Wang et al. 22-71753 (KUN-L)	**PP663149**	na	This paper
*L.praeradiosa*	China: Xinjiang	ZYY22-596 (AHUB)	**PP663150**	**PP663172**	This paper
*L.praeradiosa*	China: Xinjiang	ZYY22-570 (AHUB)	**PP663151**	**PP663173**	This paper
*L.pruinosa*	China: Inner Mongolia	Wang 20123630 (SDNU)	JX476027	na	Kou et al. (2013)
*L.pruinosa*	China: Inner Mongolia	Wang 20123278 (SDNU) Holotype	JX476028	na	Kou et al. (2013)
*L.pruinosa*	China: Inner Mongolia	Wang 20123575 (SDNU)	**PP663152**	na	This paper
*L.pruinosa*	China: Inner Mongolia	Wang 20122917 (SDNU)	**PP663153**	na	This paper
*L.pruinosa*	China: Inner Mongolia	Dong 20123276 (SDNU)	**PP663154**	na	This paper
*L.radiosa*	Czech Republic: South Moravia	Malicek 9968	ON707068	ON715664	Unpublished
*L.radiosa*	Greece	Sipman & Raus 63229 (B)	MN172452	na	Unpublished
*L.radiosa*	Sweden	Nordin 5889 (UPS)	JF703124	na	Roux et al. (2011)
*L.recedens*	Sweden	Nordin 6035 (UPS)	HQ406807	na	Owe-Larsson et al. (2011)
*L.recedens*	Portugal	Sipman 62857	MN586980	na	Sipman and Aptroot (2020)
L.subdiffractavar.rimosa	China: Xinjiang	Wang et al. 22-72975 (KUN-L)	**PP663155**	**PP663174**	This paper
L.subdiffractavar.rimosa	China: Xinjiang	ZYY22-647 (KUN-L) Holotype	**PP663156**	**PP663175**	This paper
*L.semisterilis*	China: Qinghai	Wang et al. 18-59322 (KUN-L)	MK778039	na	Zhang et al. (2020)
*L.semisterilis*	China: Qinghai	Wang et al. 18-59345 (KUN-L)	MK778042	na	Zhang et al. (2020)
*L.semisterilis*	China: Gansu	Wang et al. 22-73123T (KUN-L)	**PP663157**	**PP663176**	This paper
*L.semisterilis*	China: Gansu	Wang et al. 22-73079A (KUN-L)	**PP663158**	**PP663177**	This paper
*L.semisterilis*	China: Gansu	ZYY22-715 (AHUB)	**PP663159**	**PP663178**	This paper
*L.semisterilis*	China: Gansu	ZYY22-719 (AHUB)	**PP663160**	**PP663179**	This paper
*L.semisterilis*	China: Gansu	ZYY22-704 (AHUB)	**PP663161**	**PP663180**	This paper
*L.subdiffracta*	Russia: Republic of Altai	Frolov 178-1 (UFU)	MK347503	MK348233	Paukov et al. (2019)
*L.subdiffracta*	Russia: Republic of Altai	Frolov 178-2 (UFU)	MK347504	MK348235	Paukov et al. (2019)
*L.subdiffracta*	China: Xinjiang	ZYY22-628 (AHUB)	**PP663162**	**PP663181**	This paper
*L.subdiffracta*	China: Xinjiang	Yin A. C. & Chen H. X. 22-72347 (KUN-L)	**PP663163**	**PP663182**	This paper

**Figure 1.**
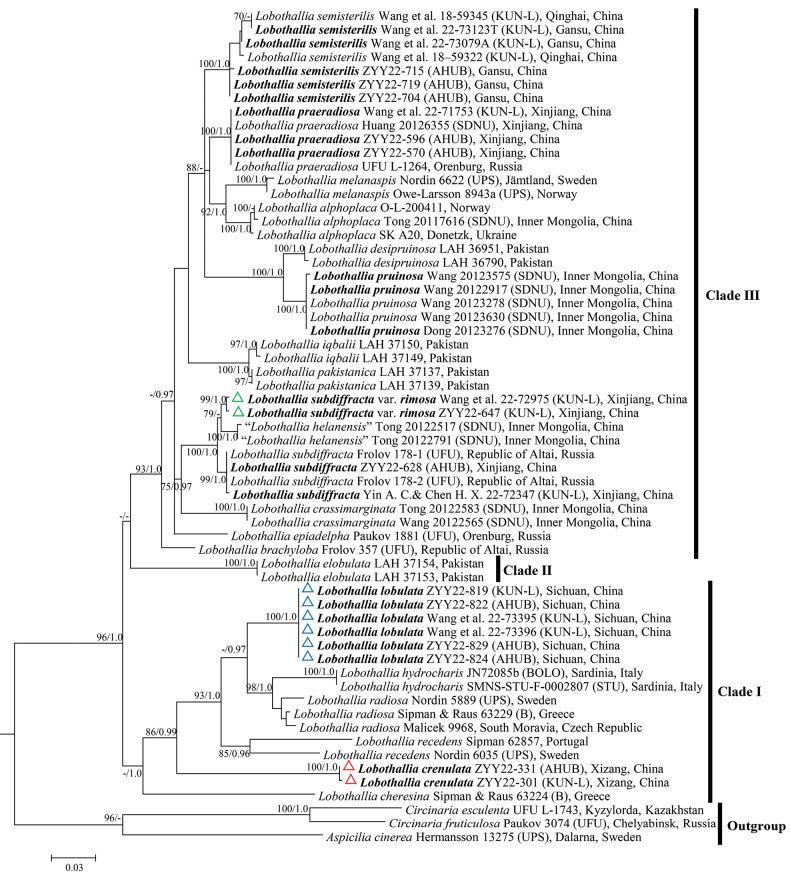
Phylogenetic tree generated from Maximum Likelihood (ML) analysis, based on the concatenated nrITS and mtSSU dataset. ML bootstrap values ≥ 70% (left) and Bayesian posterior probabilities ≥ 0.95 (right) are displayed along the branches of the tree. Newly-generated sequences are indicated in bold. The three new taxa are marked by triangles.

The two-locus phylogenetic tree showed that species of the genus *Lobothallia* fell into three main clades (Fig. 1). Our two new species, *Lobothalliacrenulata* and *L.lobulata*, formed highly supported monophyletic clades, which belonged to Clade I. *Lobothalliacheresina* is the basal species of this clade, differing from other species of this clade by its non-lobate thallus with definite cracks up to the margins and aspicilioid apothecia without prominent margin (Müller 1880; Paukov et al. 2019; Zulfiqar et al. 2022). *Lobothallialobulata* and *L.crenulata*, together with the species of *L.hydrocharis* (Poelt & Nimis) Sohrabi & Nimis, *L.radiosa* and *L.recedens* (Taylor) A. Nordin, Savić & Tibell formed a monophyletic subclade. Species of this subclade had no secondary metabolites, with the exceptions of *L.radiosa*, which has three chemotypes: chemotype *parasitica* (stictic acid), chemotype *subcircinata* (norstictic acid) and chemotype *radiosa* (without norstictic or with trace amount of norstictic acid) (Nimis and Poelt 1987; Ryan 2004; Paukov et al. 2019; Zulfiqar et al. 2022). *Lobothalliacrenulata* is the basal species of this subclade and differs from the other species by its thickly pruinose thallus with rimose upper surface and its crenate thalline margin. *Lobothallialobulata* is sister to a subclade formed by *L.hydrocharis* and *L.radiosa*, but differs in the presence of lobules at the upper surface and its lecanorine to zeorine apothecia at maturity.

Clade II comprises a single species, *Lobothalliaelobulata* Zulfiqar, Khalid & Paukov, which is easily recognised by its black prothallus, non-lobate thallus with a smooth and epruinose upper surface, lecanorine apothecia with thinly pruinose disc and the absence of secondary metabolites (Zulfiqar et al. 2022). Further research is required regarding the phylogenetic position of this clade.

Clade III consisted of several subclades and species lineages. Species of this clade were mainly distributed in Asia. Our new variety; Lobothalliasubdiffractavar.rimosa, together with samples of “*L.helanensis*” formed a sister group to *L.subdiffracta*, which was nested within Clade III. These taxa differ from other species of this clade by their non-lobate, thick and areolate thallus, rimose upper surface and absence of secondary metabolites (Magnusson 1944; Kou et al. 2013; Paukov et al. 2019). The species “*Lobothalliahelanensis*” was synonymised to *L.subdiffracta* by Paukov et al. (2019). Lobothalliasubdiffractavar.rimosa differs from *L.subdiffracta* by its lecanorine apothecia with permanent thalline margin and pruinose discs.

Nine out of the 28 species within the genus *Lobothallia* have no available gene sequences. Amongst these, secondary metabolites are only absent for *Lobothalliachadefaudiana* (Cl. Roux) A. Nordin, Cl. Roux & Sohrabi. *Lobothalliachadefaudiana* can be distinguished from our new taxa by the non-lobate thallus, with rough yellowish granules on the upper surface and immersed apothecia (Roux 1977; Paukov et al. 2019). *Lobothalliacernohorskyana* (Clauzade & Vězda) A. Nordin, Cl. Roux & Sohrabi, *L.controversa* Cl. Roux & A. Nordin, *L.gangwondoana* S.Y. Kondr., J.J. Woo & Hur and *L.lacteola* (Oxner) Şenkard., Paukov, Davydov & Sohrabi differ from the two new species by their non-lobate thallus, aspicilioid apothecia and the presence of norstictic acid (Clauzade and Vězda 1970; Roux et al. 2016; Paukov et al. 2019; Kondratyuk et al. 2020). *Lobothalliazogtii* is characterised by the brown thallus, white bordered squamules and the presence of stictic acid complex (Paukov et al. 2019). *Lobothalliaplatycarpa* shares whitish-grey and lobate thallus with the new species of *L.crenulata*, but differs in its immersed apothecia and the presence of norstictic acid (Zulfiqar et al. 2022). *Lobothalliahedinii* could potentially be confused with *L.lobulata*, but differs by its brown thallus, straight and parallel lobes and presence of norstictic acid (Magnusson 1940; Paukov et al. 2019).

### ﻿Taxonomy

#### ﻿ Lobothallia crenulata

Taxon classificationFungiPertusarialesMegasporaceae

Lun Wang & Y. Y. Zhang sp. nov.

928B1684-6853-5D78-A5D6-143BA5CB36C3

Fungal Names: FN 571927

Fig. 2A–J

##### Type.

China • Xizang Autonomous Region: Shigatse Ci., Sa′gya Co.; 29°12′01.28″N; 88°23′09.65″E; 3924 m elev.; on schist rock in a desert environment; 14 June 2022; ZYY22-301 (Holotype: KUN-L0081882!, Isotype: AHUB-00157!).

##### Diagnosis.

*Lobothalliacrenulata* is characterised by its placodioid, thickly pruinose thallus, rimose upper surface, aspicilioid to lecanorine apothecia with a crenate thalline margin, concave, black and pruinose disc and the absence of secondary metabolites.

##### Etymology.

The epithet refers to the crenate thalline margin of the apothecia.

##### Description.

**Figure 2.**
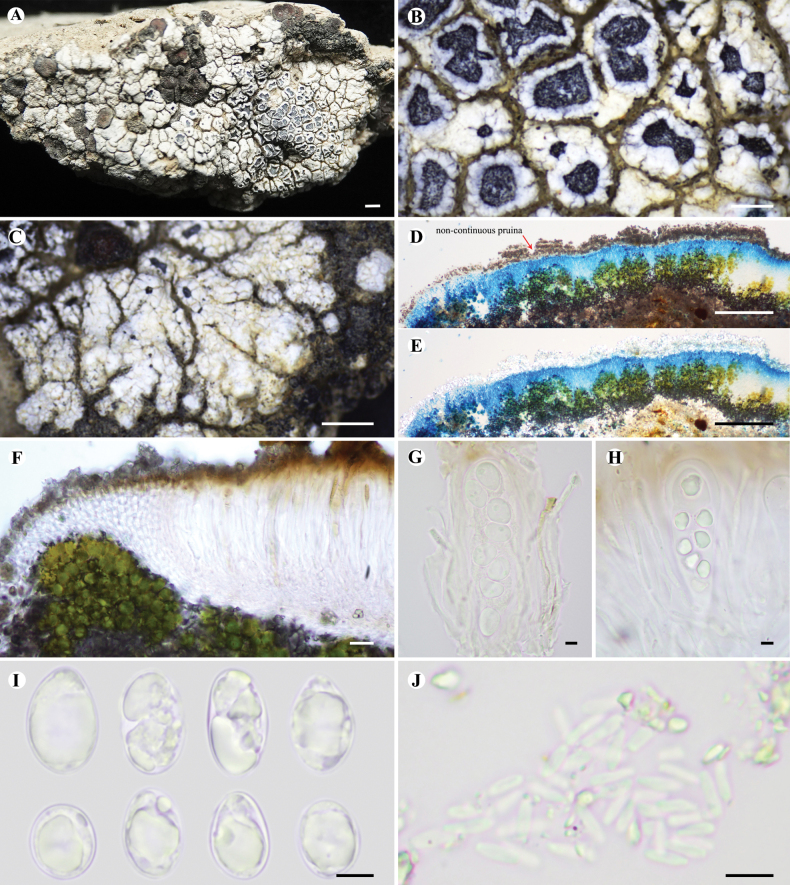
*Lobothalliacrenulata***A** thallus and apothecia **B** apothecia **C** marginal lobes **D** cross-section of thallus (LCB) **E** cross-section of thallus under polarised light (LCB) **F** vertical section of apothecia **G, H** ascus **I** ascospores **J** conidia. All sections were mounted in water, except where otherwise stated. Scale bars: 1 mm (**A, C**); 0.4 mm (**B**); 100 µm (**D, E**); 20 µm (**F**); 5 µm (**G, H, I, J**).

Thallus placodioid, circular to irregular in outline, up to 2 cm in diameter; central areoles contiguous, angular to rounded, flat to slightly convex, 0.5–2 mm wide; marginal lobes closely attached, 0.5–2 mm long, 0.2–1 mm wide, with an irregularly arranged and divided apex. Upper surface white to light grey, covered with white, thick and discontinuous pruina (see Fig. 2D). Upper cortex paraplectenchymatous, hyaline, 20–50 μm thick; epinecral layer 20–70 μm thick, consisting of dark granules (POL+, insoluble in K); algal layer discontinuous, interrupted by fungal tissue, forming separated groups, 50–150 μm high, diffuse dark granules (soluble in K), algae ca. 10–20 μm in diam.; medulla with dark brown granules (POL+, insoluble in K). Lower cortex absent.

Apothecia aspicilioid to lecanorine, numerous, 1–2 per areole, dispersed to crowded, rounded to flexuous, 0.1–1.25 mm in diam.; disc concave, black and thinly pruinose; thalline margin crenate, concolorous with the thallus, 0.05–0.125 mm wide; proper exciple inconspicuous. Hymenium hyaline, 75–120 µm high, I + blue; epihymenium 10–20 µm high, with inspersed pale brown (insoluble in K) and brown (soluble in K) granules and coarse hyaline plate-like crystals (insoluble in K); paraphyses simple, submoniliform at upper part, with 2–4 apical cells, apex thickened, 3–5 µm wide; hypothecium 30–60 μm high, hyaline, I+ blue; asci 8-spored, clavate, ***Aspicilia***-type, 70–80 × 20–30 µm; ascospores hyaline, simple, broadly ellipsoid to ellipsoid, (8.0–)11.0–13.0–14.5(–17.0) × (7.0–)8.5–9.0–10.0(–11.0) µm (n = 56), wall ca. 1.0 µm. Pycnidia uncommon; conidia bacilliform, 5–6 × 1–1.5 µm.

##### Chemistry.

K–, C–, KC–. No substances were detected by TLC.

##### Distribution and ecology.

This new species grows on calcareous schist rocks at elevations of 3924–4304 m in Xizang Autonomous Region, China.

##### Notes.

The new species is similar to *Lobothalliaiqbalii* Zulfiqar, Khalid & Paukov and *L.pakistanica* Razzaq, Fayyaz, Khalid & Afshan in its placodioid thallus, white to light grey upper surface and the absence of secondary metabolites. *Lobothalliaiqbalii* differs in its lecanorine apothecia with plane to convex disc and an entire and thick thalline margin (Zulfiqar et al. 2022). *Lobothalliapakistanica* differs in its rarely cracked central areoles, thinner epinecral layer (8–16 µm), slightly concave to flat, rarely pruinose disc and the absence of thalline margin (Zulfiqar et al. 2022). *Lobothalliasubdiffracta* shares some features with *L.crenulata*: rimose and pruinose thallus. However, *L.subdiffracta* differs in its grey thallus with thinner and uneven pruina and its non-lobate thallus (Magnusson 1944; Kou et al. 2013; Paukov et al. 2019). Another taxon, *Lobothalliapruinosa*, also has a placodioid and pruinose thallus and pruinose discs, but differs from *L.crenulata* in its entire thalline margin and the presence of norstictic and constictic acids (Kou et al. 2013).

##### Additional specimens examined.

China • Xizang Autonomous Region: Shigatse Ci., Dingri Co., along road G219; 28°35′10.03″N, 87°3′42.56″E; 4304 m elev.; on weathered schist rock; 16 June 2022; ZYY22-331 (KUN-L0081892, AHUB-00187).

#### ﻿ Lobothallia lobulata

Taxon classificationFungiPertusarialesMegasporaceae

Lun Wang & Y. Y. Zhang sp. nov.

8D407007-CB9E-5302-959F-83D804258847

Fungal Names: FN 571928

Fig. 3A–K

##### Type.

China • Sichuan Prov.: Ganzi Tibetan Autonomous Prefecture, Xinlong County, along road G227; 31°25′52.77″N, 100°8′52.04″E; 3296 m elev.; on rock; 11 July 2022; ZYY22-819 (Holotype: KUN-L0082392!; Isotype: AHUB-00673!).

##### Diagnosis.

The species *Lobothallialobulata* is characterised by its conspicuously radiate marginal lobes, pruinose upper surface with lobules, the aspicilioid apothecia when immature, lecanorine to zeorine at maturity with epruinose, shiny discs and the absence of secondary metabolites.

##### Etymology.

The epithet refers to its lobules along the upper surface.

##### Description.

Thallus placodioid, circular in outline, up to 3 cm in diameter, tightly adnate to the substratum; central areoles contiguous, angular to irregular, plane to slightly convex, 0.3–1 mm across; marginal lobes radiate, plane, 1–5 mm long, 0.5–1 mm wide, ca. 0.3 mm thick, apex rounded, irregularly divided, usually with a black rim. Upper surface light grey to greyish-olive, lightly and discontinuously pruinose, pruina on the apex of lobes thicker than the centre. Lobules common, 0.1–0.3 mm, divided, heavily pruinose. Upper cortex paraplectenchymatous, even, ca. 30 μm thick, filled with pale brown (insoluble in K) and dark brown (partly soluble in K) granules; epinecral layer 10–20 μm thick, containing dark granules when pruina is present (POL+, insoluble in K); algal layer discontinuous, interrupted by fungal tissue, forming separated groups, 50–150 μm high, containing black substance (soluble in K), algae cells ca. 5–10 μm in diam.; medulla filled with black substance (POL+, insoluble in K). Lower cortex absent.

Apothecia aspicilioid when immature, lecanorine to zeorine at maturity, common, initially 1–2 per areole, usually one per areole, scattered to slightly grouped, adnate, rounded, 0.5–1 mm in diam.; disc brown to brownish-black, shiny, epruinose, concave at first, plane at maturity; thalline margin entire, ca. 0.1 mm wide, slightly pruinose or epruinose, cortex identical with upper cortex (POL–), 30–50 μm thick; proper exciple conspicuous in mature apothecia, 20–150 μm thick. Hymenium hyaline, I+ blue, 100–120 μm high; epihymenium 5–10 μm thick, with pale brown (insoluble in K) and brown (soluble in K) granules; paraphyses simple, moniliform at upper part, with 3–6 cells, apex thickened, 4–6 µm wide; hypothecium 25–50 μm thick, hyaline, I+ blue; asci 8-spored, clavate, ***Aspicilia***-type, 70–80 × 15–20 µm; ascospores hyaline, simple, broadly ellipsoid, (9.0–)10.5–11.5–12.5(–13.0) × (7.0–)8.0–8.5–9.5(–10.0) µm (n = 64), wall ca. 1.0 µm. Pycnidia common, convex, ostioles dark brown, shiny; conidia hyaline, bacilliform, 5–6 × ca. 1 μm.

**Figure 3.**
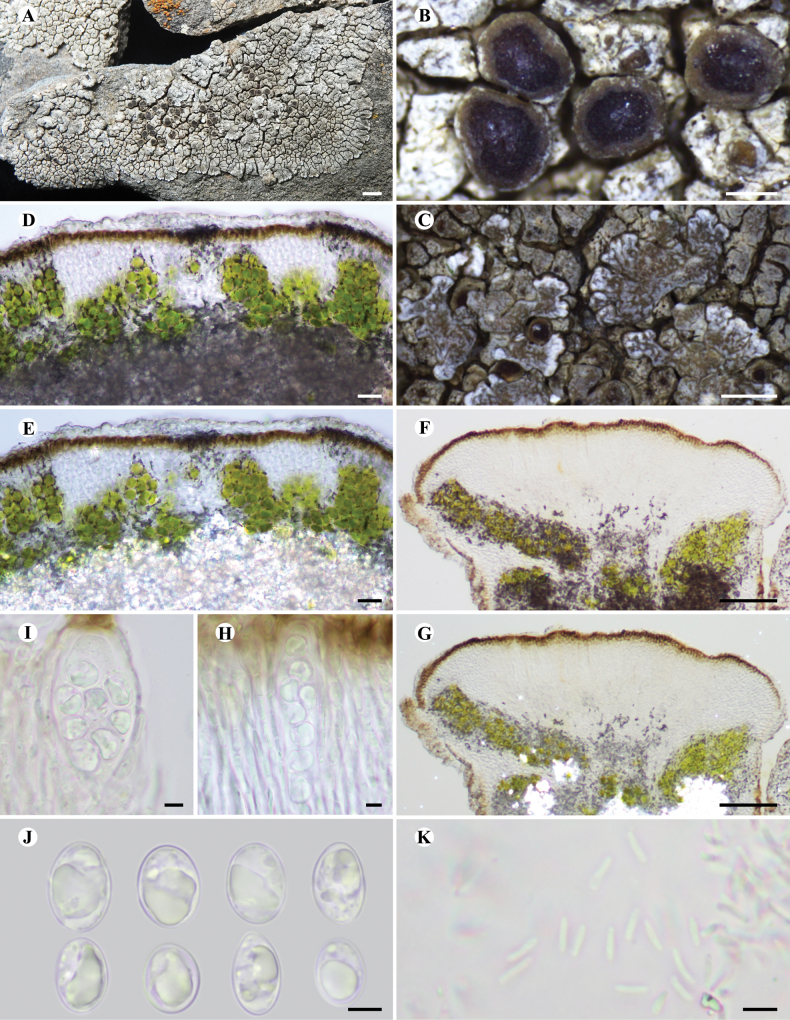
*Lobothallialobulata***A** thallus and apothecia **B** apothecia **C** lobules **D** cross-section of thallus **E** cross-section of thallus under polarised light **F** vertical section of apothecia **G** vertical section of apothecia under polarised light **H, I** ascus **J** ascospores **K** conidia. All sections were mounted in water, except where otherwise stated. Scale bars: 2 mm (**A**); 0.4 mm (**B**); 1 mm (**C**); 20 µm (**D, E**); 50 µm (**F, G**); 5 µm (**H, I, J, K**).

##### Chemistry.

K–, C–, KC–. No substances were detected by TLC.

##### Distribution and ecology.

This new species grows on exposed calcareous rocks at elevations of 3262–3296 m elev. in Sichuan Province, China.

##### Notes.

This species shares morphological features with the closely-related *Lobothalliaradiosa*: both have placodioid thallus, areolate in central parts and conspicuously radiate marginal lobes. *Lobothalliaradiosa* has three chemotypes: *parasitica* with stictic acid, *subcircinata* with norstictic acid and *radiosa* with or without a trace amount of norstictic acid (Ryan 2004; Reyim et al. 2012; Paukov et al. 2019). The new species shares the chemotype of some specimens of *radiosa*, but differs in the presence of lobules, the aspicilioid apothecia when immature, lecanorine to zeorine at maturity and in its phylogenetic position. *Lobothalliahydrocharis* also has a placodioid thallus with secondary metabolites absent, but differs by its aspicilioid apothecia with black and matt discs and its distribution, which is restricted to Sardinia, Italy (Nimis and Poelt 1987; Nimis 2016; Nascimbene et al. 2023).

##### Additional specimens examined.

China • Sichuan Prov.: Xinlong Co., along road G227; 31°25′53″N, 100°8′53″E; 3282–3296 m elev.; on rock; 11 July 2022; ZYY22-822 (KUN-L0082395, AHUB-00676), ZYY22-824 (KUN-L0082397, AHUB-00678), ZYY22-829 (KUN-L0082402, AHUB-00683) • Shadui Vi.; 31°25′52″N, 100°8′54″E; 3262–3263 m elev.; on limestone rock; 11 July 2022; Li S. Wang et al.; 22-73395 (KUN-L0087873), 22-73396 (KUN-L0087874).

#### ﻿ Lobothallia subdiffracta var. rimosa

Taxon classificationFungiPertusarialesMegasporaceae

Lun Wang & Y. Y. Zhang var. nov.

17100411-9150-5AB3-9DB8-994660D3F1F2

Fungal Names: FN 571929

Fig. 4 A–J

##### Type.

China • Xinjiang Uygur Autonomous Region: Hami Ci., Balikun Co.; 43°41′39″N, 92°17′48″E; 2031 m elev.; on rock; 04 July 2022; ZYY22-647 (Holotype: KUN-L0082221!, isotype: AHUB00501!).

##### Diagnosis.

Lobothalliasubdiffractavar.rimosa is characterised by its areolate thallus with slightly radiated marginal areoles, rimose and white pruinose upper surface, lecanorine apothecia with black and pruinose discs, its crenate thalline margin when immature and entire at maturity and the absence of secondary metabolites.

##### Etymology.

The epithet refers to the rimose upper surface.

##### Description.

Thallus areolate, usually circular in outline, up to 4 cm in diam., 2–5 mm thick, central areoles continuous, angular and slightly convex, 0.5–2.5 mm across, marginal areoles slightly radiate with a rounded apex. Upper surface greyish to clay coloured, rimose and pruinose. Upper cortex paraplectenchymatous, uneven, (25.0–)36.5–53.5–70.5(–85.0) μm (n = 20) thick, upper part brownish, insoluble in K; epinecral layer uneven, (10.0–)21.5–45.5–69.0(–95.0) μm (n = 30) thick, containing dark brown granules (POL+, insoluble in K); algal layer discontinuous, interrupted by fungal tissue, forming algal stacks, 100–200 μm high, with dark granules (partly soluble in K), algae 8–15 μm in diam.; medulla containing black substance (POL+, insoluble in K). Lower cortex absent.

**Figure 4.**
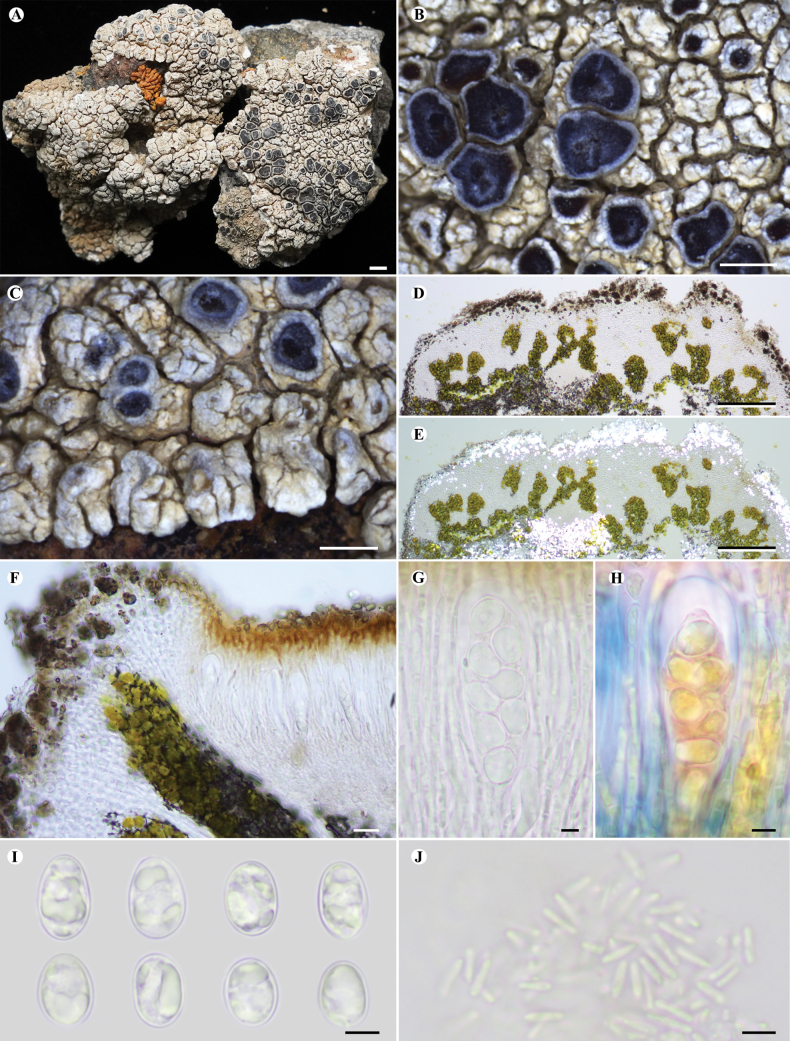
Lobothalliasubdiffractavar.rimosa**A** thallus and apothecia **B** thalline margin crenate when immature and entire at maturity **C** slightly radiate arrangement of marginal areoles **D** cross-section of thallus **E** cross-section of thallus under polarised light **F** vertical section of apothecia **G** ascus **H** ascus (Lugol’s solution) **I** ascospores **J** conidia. All sections were mounted in water except where otherwise stated. Scale bars: 2 mm (**A**); 1 mm (**B, C**); 100 µm (**D, E**); 20 µm (**F**); 5 µm (**G, H, I, J**).

Apothecia lecanorine, numerous, initially 1–2 per areole, then one per areole, dispersed to grouped, rounded, (0.2–)0.7–1.5(–2) mm in diam.; disc slightly concave to plane, matt, black, pruinose; thalline margin 0.1–0.15 mm wide, crenate when young, entire with age, pruinose, concolorous with upper surface, cortex identical with upper cortex (POL+), 40–75 μm thick; proper exciple inconspicuous. Hymenium hyaline, I+ blue, 100–125 μm high; epihymenium 5–15 μm thick, with pale brown (insoluble in K), brown (soluble in K) granules and hyaline plate-like crystals (insoluble in K); paraphyses simple to rarely anastomosed, submoniliform to moniliform at the upper part, with 3–5 cells, apex thickened, 4–6 µm wide; hypothecium 50–60 μm thick, hyaline, I+ blue; asci 8-spored, clavate, ***Aspicilia***-type, 60–80 × 20–30 µm; ascospores hyaline, simple, broadly ellipsoid, (8.0–)10.0–11.0–12.5(–13.0) × (7.0–)7.0–8.0–9.0(–10.0) µm (n = 50), wall ca. 1.0 µm. Pycnidia common, with punctiform ostiole, dark brown; conidia hyaline, bacilliform, 5–7(–8) × ca. 1 μm.

##### Chemistry.

K–, C–, KC–. No substances were detected by TLC.

##### Distribution and ecology.

This species grows on exposed calcareous rocks at elevations of approximately 2000 m in Xinjiang Uygur Autonomous Region, China.

##### Notes.

Lobothalliasubdiffractavar.rimosa, L.subdiffractavar.subdiffracta and “*L.helanensis*” were sympatric in north-western China and neighbouring regions and also phylogenetically closely interrelated (Kou et al. 2013; Paukov et al. 2019). “*Lobothalliahelanensis*” was previously synonymised with *L.subdiffracta*, because both shared the morphological characters of non-lobate thallus and apothecia with incised margins, with similar sequences in the ITS and mtSSU regions (Paukov et al. 2019). Our materials differ from both var. subdiffracta and “*L.helanensis*” by their characters of lecanorine apothecia, permanent thalline margin, pruinose discs and the slightly radiate marginal areoles. Therefore, we treat these specimens as a new variety within *Lobothalliasubdiffracta*. Table 2 presents a brief comparison of these taxa. Another taxon *Lobothalliarecedens* may be confused with L.subdiffractavar.rimosa due to its thick, areolate, non-lobate thallus and the absence of secondary metabolites. However, the former differs in its densely clustered apothecia (3–6 per areole) and its shorter conidia 3–5 × ca. 1 μm (Paukov et al. 2019; Cannon et al. 2023; Martellos et al. 2023).

**Table 2.** Comparison between Lobothalliavar.subdiffracta, “*L.helanensis*” and L.var.rimosa.

Character	L.var.subdiffracta	“*L.helanensis*”	L.var.rimosa
Thallus form	areolate	areolate	areolate, marginal areoles slightly elongate
Apothecia form and size (mm)	aspicilioid, disc 0.2–0.4(–1.5 in our newly collected materials) in diam.	aspicilioid, 0.5–1.3(–2) in diam.	lecanorine, (0.2–)0.7–1.5(–2) in diam.
Disc	epruinose	epruinose	pruinose
Habit (substratum)	siliceous rock	calcareous rock	calcareous rock
References	Magnusson (1944); Paukov et al. (2019)	Kou et al. (2013)	This paper

##### Additional specimens examined.

Lobothalliasubdiffractavar.rimosa. China • Xinjiang Uygur Autonomous Region: Hami Ci., Balikun Co., along road G335, 43°41′35.55″N, 92°17′46.81″E, 2036 m elev., on limestone rock, 04 July 2022, Li S. Wang et al. 22-72975 (KUN-L0087453).

Lobothalliasubdiffractavar.subdiffracta. China • Xinjiang Uygur Autonomous Region: Fukang Ci., Chengguan Vi.; 44°09′36.52″N, 87°58′42.00″E; 500–600 m elev.; on sandstone; 04 July 2022; Yin A. C. & Chen H. X.; 22-72347 (KUN-L0086973) • Turpan Ci., Tuokexun Co.; 43°06′15.85″N, 87°34′52.51″E; 2473 m elev.; on rock; 02 July 2022; ZYY22-628 (KUN-L0082202, AHUB-00482).

“*Lobothalliahelanensis*”. China • Inner Mongolia: Bayan Hot Vi., Helan Mountain; 1500–2000 m elev.; on rock; 19 Aug 2011; Wang H. Y. 20122708; Kou X. R. 20123833, Wang P. M. 20123198, Dong D. B. 20123040 (SDUN).

### ﻿Key to the species of *Lobothallia* in China

**Table:** 

1	Thallus areolate, margins not lobate	**2**
–	Thallus placodioid, margins distinctly lobate	**4**
2	Thallus whitish with grey tinge, upper surface not farinose, with definite cracks up to the margin. Orbicular specimens with marginally radially elongated cracks, given the thallus a placodioid-like appearance. Apothecia without prominent thalline margin. Secondary metabolites absent or stictic/norstictic acid present	***Lobothalliacheresina***
–	Thallus pruinose and rimose, not cracked. Apothecia with prominent thalline margin. Secondary metabolites absent	**3**
3	Thallus light to dark grey to olive grey. Apothecia aspicilioid to lecanorine, with an epruinose disc	**Lobothalliasubdiffractavar.subdiffracta**
–	Thallus greyish to clay coloured. Apothecia lecanorine, with a pruinose disc	**Lobothalliasubdiffractavar.rimosa**
4	Terricolous, pycnidia prominent, sometimes protruding apothecia-like	***Lobothalliasemisterilis***
–	Saxicolous, pycnidia immersed to slightly convex with depressed or punctiform ostiole	**5**
5	Secondary metabolites absent	**6**
–	Secondary metabolites present	**7**
6	Lobules present at the upper surface, discs shiny and epruinose, thalline margin entire	***Lobothallialobulata***
–	Lobules absent, discs matt and pruinose, thalline margin crenate	***Lobothalliacrenulata***
7	Norstictic acid present	**8**
–	Norstictic acid absent, but stictic acid present. Thallus brown, lobes with definite deep cracks forming a reticulate pattern in exposed habitat	***Lobothalliazogtii***
8	Thallus epruinose	**9**
–	Thallus pruinose	**11**
9	Thallus loosely attached to the substratum (sometimes removable intact). Central areoles strongly swollen, bullate. Lobes often strongly convex to almost cylindrical	***Lobothalliaalphoplaca***
–	Thallus closely attached to the substratum. Central areoles plane to convex or uneven, not bullate. Lobes plane to moderately convex	**10**
10	Lobes 1–2 mm long, closely attached, not overlapping. Upper surface grey, sometimes tinted ochraceous or rosy. Apothecia with thick thalline margin, 0.2–0.5 mm wide. On calcareous rocks	***Lobothalliacrassimarginata***
–	Lobes 3–6 mm long, loosely attached, overlapping. Upper surface green grey to orange brown. Apothecia with narrower thalline margin (less than 0.3 mm wide). On siliceous rocks	***Lobothalliapraeradiosa***
11	Thallus brown, lobes strongly convex, simple to dichotomous, with straight and parallel margins	***Lobothalliahedinii***
–	Thallus whitish-grey to brownish-grey or grey, lobes flat or moderately convex	**12**
12	Lobes flat, 1–3 mm long. Constictic acid present	***Lobothalliapruinosa***
–	Lobes moderately convex, 3–5 mm long, with darkened tips. Constictic acid absent	***Lobothalliaradiosa***

## Supplementary Material

XML Treatment for Lobothallia crenulata

XML Treatment for Lobothallia lobulata

XML Treatment for Lobothallia subdiffracta var. rimosa

## ﻿Additional information

### Author ORCIDs

Yanyun Zhang https://orcid.org/0000-0002-0902-5066

Xinyu Wang https://orcid.org/0000-0003-2166-6111

### Data availability

All of the data that support the findings of this study are available in the main text.
